# Emergency Preparedness and Management at the University of L’aquila (Central Italy) and the Role of Students’ Associations in the April 6th 2009 Earthquake

**DOI:** 10.1371/currents.dis.5df8f1902f10be8920342035c77c14e3

**Published:** 2017-01-12

**Authors:** Michele Magni, Rita Fraboni, Fausto Marincioni

**Affiliations:** Department of Life and Environmental Sciences, Università Politecnica delle Marche, Ancona, Italy; Health and Safety Office, Università Politecnica delle Marche, Ancona, Italy; Department of Life and Environmental Sciences, Università Politecnica delle Marche, Ancona, Italy

## Abstract

Introduction: On April 6th 2009 an earthquake of Mw=6.3 hit the historical downtown of L’Aquila and its hinterland causing more than 300 fatalities and severe damage to private and public buildings. At the time, the University of L’Aquila represented a major source of employment and income for the city. The earthquake impacted both the facilities and the administrative, financial and patrimonial activities of the university, bringing into the open the tendency – widespread in Italy – to rely on adaptive tactics rather than on strategic pre-disaster plans. This paper investigates the university’s emergency preparedness and response capability and  the strategies adopted to restore the education activities as well as avoid students migration to other universities. In addition, emphasis is placed on the role played by Student Associations in pre and post-disaster phases, and how students perceived the activities performed by these associations.

Methods: To achieve this goal, it was undertaken: i) qualitative evaluation to assess the impact of earthquake on services and facilities of the university, the emergency preparedness and the measures adopted to face the emergency, ii) survey on the role played by Student Associations, both in emergency preparedness and response, according to students’ perception; iii) quantitative analysis to measure changes in the enrollment trend after the earthquake, and how university policies could curb students’ migration.

Results: The policies adopted by the University allowed to diminish students’ migration; however, the measures taken by the university were based on an ad hoc plan as no emergency and continuity plans were prepared in advance. Similarly Student Associations got involved more in restoration activities than in emergency preparedness and risk awareness promotion.

Discussion: Greater awareness and involvement are essential at each level (administrators, faculties, students) to plan in advance for an adverse scenario and to make important steps forward in understanding and embracing a culture of safety. The present paper is starting point for future research to deepen the emergency preparedness of Universities and the role that Student Associations may play to support and spread such a culture of safety.

## Introduction

At 3:32 a.m. (UTC+1) on 6 April 2009 an earthquake of Mw=6.3 hit the historical downtown of L'Aquila and its hinterland. The mainshock was anticipated by a long lasting seismic cluster (Ml<4.0) that begun in the second half of December 2008. Luckily, two major shakes of Ml=3.9 and Ml=3.5 developed few hours before the April 6^th^ mainshock, alerted the population and induced part of them to flee the buildings and spend the night outside 1 ^,^ 2. The earthquake caused 308 dead, 1500 injured and 63000 displaced. Among the dead, 55 were university students, of which 8 died in the collapse of a university dormitory. Had the earthquake occurred in daytime, the people involved in the collapse of ancient public buildings would have been probably much higher. The high seismic risk in Italy depends not only on the frequency and the intensity of the earthquake hazard, but also on the vulnerability of the built environment. The Italian territory is dotted by many ancient buildings liable to collapse during earthquake 1 ^,^ 2 ^,^ 3 ^,^ 4 ^,^ 5 ^,^ 6. The government agency responsible for promotion and coordination of disaster risk reduction activities in Italy is the Department of Civil Protection (DPC), which was established by Law 225/92, recently amended by Law 100/2012. Other and more specific framework laws (e.g. L.D. 81/2008 8, M.D.10 March 1998 7) define the responsibilities and duties of certain public agencies, including the universities, in terms of emergency planning and risk reduction. In the specific case of public universities, the Law gives to their government bodies (e.g. the chancellor, the academic senate, and the board of directors) a certain degree of autonomy to decide strategies for disaster risk reduction, including the staffing and organization of an internal emergency management group. Indeed, emergency planning beside improving safety and security of students, is a moral obligation of universities, which also safeguards the execution of their institutional scope, namely higher education and professional training activities. The inability to provide basic services like courses, exams, graduation, research, after a disaster, may fatally impact a university reputation and financial status. This becomes particularly important, when the university plays a key role in the economy of the local community. At the time of the earthquake, the University of L’Aquila had 23000 students enrolled across its various graduate and undergraduate degree programs; the student population was almost one third of the total number of residents of L’Aquila 9. Hence, the university was a major source of employment and income for the city and its surroundings, as well as a fundamental drive for cultural and social activities of the city. The negative effects endured by the university bore direct consequences on the city, the inhabitants and particularly the students. In this scenario, the students are not just patrons of the university, but also part of the social fabric of the city; thus, students should be involved in the emergency planning processes of both the city and the university. Such involvement happened only in minimal part in the 2009 earthquake. In Italy, as in many other parts of the World, students take part of the university governance process through the Student Associations. These organizations are directly managed by the students and are dedicated to improve all the aspects of students’ life in higher education. Their scope ranges from safeguarding students’ rights (e.g. welfare and representation) to promote social, political and cultural initiatives. Concurrently, Student Associations are an important interface between the university and the local community, contributing to the civic debates and processes 10 ^,^ 11. The University of L’Aquila was not an exception to this, and four major student organizations were active at the time of the earthquake: the “Azione Universitaria” (rightist political view), “Lista Aperta” (catholic oriented), “Unione degli Universitari” (leftist political view), and “Modus” (non-partisan). All the Srudent Assoctions took part to the University politics with initiatives on the “right to education” and student safety and security. Analyzing the impact of the April 6^th^ 2009 earthquake on the University of L’Aquila both in terms of facilities and business continuity, this study assesses the emergency reparedness and response capability of such university, and the strategies adopted to restore the education activities and avoid students migration to other universities. In addition, emphasis is placed on the role played by Student Associations during pre and post-disaster phases, as well as how the student population perceived the activities performed by these associations.

## Emergency management in universities

Over the past years various disasters affected universities and higher education institutions worldwide. For example, Hurricane Katrina's impacted Tulane and Louisiana State Universities (August 2005). The Midwest seismic event (April 2008). Those events determined economic losses to universities both in terms of structural damages and disruption of academic activities. Indeed, comprehensive pre-disaster planning and preparation can considerably reduce the negative effect of extreme events 12. The US Federal Emergency Management Agency (FEMA) has financed the Disaster Resistant University (DRU) program, in order to develop planning strategies for vulnerability reduction in american universities 13 ^,^ 14. In Canada, universities are not held directly responsible for emergency management; emergency planning and management is coordinate with local authorities 15. In England, Easthope and Eyre 16 published a manual entitled “Planning for and Managing Emergencies: A Good Practice Guide for Higher Education Institutions (HEIs). The authors made a distinction between the emergency planning and the business continuity management. Special attention is dedicated to training, drills and exercises, and emergency communications, with the latter having a key role in the planning processes 16 ^,^ 17. In Italy, the issue of safety and security in Universities was tackled in 2007 by the Italian Universities Chancellors’ Permanent Conference (CRUI), which commissioned a thorough investigation of how emergency management regulations are applied within the public university precincts. The study showed that a vast majority of the Italian universities has developed, within their organizational structure, a Health and Safety Office (HSO) to which are delegated emergency management responsibilities. However, CRUI found that 47.2 % of the universities do not perform emergency planning activities systematically. Therefore, even if the Italian universities started to incorporate safety and security strategies in its official procedures, many steps forward have to be done to turn theory into practice 18. Some hindrances also arise from the Italian law framework on emergency management for universities, which is strictly connected with workplace health and safety 7 ^,^ 8 and it is only focused on the immediate response to minor operational incident (in contained areas or within a classroom or laboratory). The March 10th 1998 Decree of the Italian Ministry of Interiors defines the criteria about the fire control and workplace accident management, drafting evacuation and the fire brigades alarm procedures. As a consequence, disaster prevention or business continuity management is not really contemplated in Italian university, and there is a widespread underestimation of risks by the local emergency managers 18.

## University Student Associations and emergency preparedness: a neglected nexus

In spite of the different forms of configuration, management, financing and scope of Student Associations in different parts of the World, a common purpose of these organizations is representing students’ prerogatives to enhance their studying experience (e.g. asking for better school services, curriculum or educational funding). Many Student Associations are nonpartisan, yet such organizations are catalysts of students' activism and have greatly influenced political debates on both local and global issues. For example, in the United States, France, United Kingdom, and many other countries, the political vehemence of Student Associations reached a high in the 1960s and 1970s during the protests and strikes to end the war in Vietnam and against the invasion of Cambodia 19 ^,^ 20. In the 1980s new subject matters, such as equal opportunities, feminism, environmentalism and humanitarianism, softened the political activism of Student Associations, yielding attention to more local issues of tuition fees or student representation in college governance 21. In Italy most Student Associations have a declared and deep-seated political characterization, and are organized into bodies with a hierarchical structure emulating the country’s political administrative layout. Although the first appearance of organized associations of university students in Italy can be dated back to the 1920, during the Fascist era, it was only after World War II that these associations became truly representative of the whole national students’ community. Indeed, the contestation years of the 1960s and 1970s produced protests and riots activities in Italy too. The ensuing social and civil developments of Italy, weakened the militant components of Italian Student Associations, which by the early 1990s were completely engaged in nonviolent debates. Nowadays, these associations confederated in lists coalescing into three major political views; conservative, catholic-moderate, and liberal-progressive. All these associations operate at the local level electing the “Student Council” which has propositional functions at the Faculty Council, Academic Senate and the University’s Boards of Directors. They also partake into the “National Council of University Students,” a permanent panel which select representative to the “National University Council” (the advisory board of the ministry of education that composed by university’s rectors, faculties, students and employees). Beside these nationwide and chartered Student Associations there exist other, more independent and goal oriented, student organizations. Their scope and inspiring foci can be very different one another, ranging from specific cultural interests, to international student exchange programs, including volunteer services. Unquestionably, Student Associations, in spite of their scope, national vs. local, or their objectives, partisan vs. nonpartisan, are an invaluable resource for the university on a variety of purposes, including students' safety and security. Yet, this resource is most often completely untapped in terms of disaster prevention and preparedness activities, especially for increasing risk awareness and knowledge transfer. This appear to be true in Italy as well as in many other countries; as a matter of fact, no consistent information about a systematic engagement of Student Associations in disaster risk reduction was found. The potential for this nexus is remarkable, both for developing a culture of safety with younger generations, and for boosting universities’ emergency planning and procedures. Involving students in the campus' safety and security programs, would increase their awareness of potential hazards and risks in their locale, and train them with protective behaviors and emergency procedures. Gaining students' attention on such initiatives should not be too challenging, considering the high number of enrollment of students (especially undergraduates) in hazards and disasters related degree programs and courses 22.

## Student Associations in L'Aquila

Few days after the earthquake, the National Council of Italian Students (Student Council) unanimously adopted a motion to ask the National Government for prompting the reconstruction of the University of L'Aquila and the restoration of its services. More specifically, the National Council asked the Government to rebuild an high-tech campus with first-class antiseismic standard and to exempt the students from University fees. The Council also led an initiative, named UNILIBER, to gather academic books for the University of L'Aquila in collaboration with the National Civil Protection, Rai-Radio TRE (program Fahrenheit) and the L’Aquila section of the National Youth Corp of Scouts (CNGEI). Since the days after the main shock, the democratic left wing list named “Union of University Students” (U.D.U), which had just gained the majority of seats in any representative local board at the University of L'Aquila, set up an headquarter office inside a container situated in a temporary tents camp close to the College of Natural Science in the village of Coppito. The U.D.U. kept offering logistic assistance to the students residing in the tents camp during the whole emergency. Another volunteer Student Associations named LARES, from the University of Perugia (Central Italy), joined U.D.U. to help managing students' accommodations in the tent camps of Coppito, coordinating also an information front desk inside the college of Sciences.

## Case study

The University of L’Aquila was founded in 1952, and became a full-fledged Italian State University in 1982 academic year. The University is composed of 9 Colleges (Biotechnologies, Economics, Engineering, Humanities, Medicine and Surgery, Psychology, Mathematical Physical and Natural Science, Education Science, Sport Science), 18 Departments and 2 Centre of excellence for research. At the time of the earthquake, the University had 3 campuses within the municipality of L’Aquila (Centro, Coppito, Roio), and others campuses in the neighboring municipalities (Sulmona, Avezzano and Celano). For teh academic year 2008/09, the University offered a broad choice of degree programs and courses: 41 bachelor degrees, 43 Master degrees, 24 Ph.D., 40 specialization schools. The faculty body was composed of 665 persons. During the 2007/08 academid year, on a total of 72000 people living in L'Aquila, 27168 were students. Of these students 37% came from L’Aquila and province; 32% from others Italian provinces; 28% from others provinces of Abruzzo Region; and 3% from abroad (University of L’Aquila Statistics Observatory). According to the Italian Universities templates for governance 32, the University of L’Aquila is an institution with legal personality and teaching, scientific, organizational, financial and accounting autonomy. The government bodies for guidance and control are: the Rector, the Academic Senate and the Boards of Directors 23. The Rector, the legal representative of the University, promotes and coordinates the strategies expressed by the Academic Senate and the Boards of Directors. The Academic Senate performs procedures, planning, coordination and monitoring of teaching and research activities. The Board of Directors manages and controls the administrative, financial and patrimonial activities of the University. In order to reach its institutional goals, the University fields specific facilities: Colleges, Departments and service centers. The Colleges support and manage the education; the Departments coordinate and manage the research activities; the service centers have general competences. The education services and the research activities of the L’Aquila University are almost entirely dependent on State funding (more than 80%). Others funding sources are students’ fees (about 15%) and self-financing obatined by selling services to the private and public sector. Also relevant is the participation of the Student Associations in the government bodies.

## Research questions

As discussed in the previous chapters, the Italian universities lack specific regulations on disasters recovery and business continuity mostly because the law framework is limited to standard procedures for minor incidents and emergency management in the workplace. In addition, the tendency to underestimate the risk induces universities to neglect emergency planning activities and drills. The L’Aquila earthquake represented am unusual case study as it struck an area tightly bonded with socio-cultural activities and incomes generated by the university. Furthermore, none of the seven largest earthquakes which hit the Italian peninsula in the last 50 years occurred as close to a major university campus as the one that struck L’Aquila. These circumstances led to the following questions:

RQ1: How did the earthquake affect the University services and facilities?

RQ2: How was the University prepared to face such an emergency?

RQ3: Which measures did the University adopt to recover after the earthquake?

RQ4: How did the enrollment trend change after the earthquake?

RQ5: How did the students evaluate the role of Student Associations during the pre and post disaster phases?

## Research design and data collection

This research combines multiple sources of evidences 24 and qualitative and quantitative analyses. Considering the first set of research questions, qualitative analysis aims at clarifying the earthquake impact on the University services and facilities, the emergency preparedness and the measures adopted to face the emergency (RQ1, RQ2, RQ3). To this purpose it was undertaken an evaluation of the various statistics published by the MIUR, the University of L’Aquila, and the newspaper articles. Quantitative analysis was applied to measure change in the enrollment trend (RQ4) and to assess students’ perception about the role that Student Associations played in the emergency preparedness and response (RQ5). Data published by MIUR were analyzed to assess the changes in enrollment trend, while a structured questionnaire administered to the students of the University of L’Aquila provided information to answer RQ5 and RQ6. The survey was carried out 3 months after the earthquake, adopting a non-probability convenience sampling 25. Students were approached at the campus of the college of Science (Coppito) in tents camp hosting the students coming from outside L’Aquila. The questionnaire was delivered with a drop-off pick-up methodology in order to reduce non-coverage error and possible sample bias. In addition, this methodology provides opportunities to gain experiential insights by having face-to-face contact with respondents 26 ^,^ 27. The questionnaire was anonymous and contained statements based on a five-items Likert Scale (1. Strongly disagree, 2. Disagree, 3. Uncertain, 4. Agree, 5. Strongly agree).

## Results

The results of the study are presented in the following subsections:

- University's services and facilities

- Emergency preparedness

- Recovery policies

- Enrollment trends

- Questionnaire

## University services and facilities

The report of the structural engineering task force of the University of L’Aquila, which surveyed the status of facilities within the university precincts, revealed considerable damages 28. As a matter of fact, many buildings were highly vulnerable historical edifices. Indeed, many others were modern concrete frame structures with high seismic resistance. The historical buildings were affected by total or partial collapses; for example, the College of Literature (located in Palazzo Camponeschi), the College of Humanities (Palazzo Porcinari), the Rectorate and the administrative offices (Palazzo Carli), as well as the Conventions Center of San Basilio had been seriously damaged. The modern buildings (Engineering, Economics and Medicine) survived the shakes undamaged, while the College of Sciences reported very minor damages 28. The buildings stock was immediately declared unusable, teaching was suspended and many services interrupted. The students' dormitory “casa dello studente” (the collapse of which caused 8 fatalities) and the various student lounges in Coppito were also closed. The emergency teams of the University were not on duty at the time of the earthquake and never activated. Except for the dormitories, which are under the jurisdiction of the Regione Abruzzo, the university is closed at night. Therefore the rescue operations were coordinated by the Italian National Department of Civil Protection with the support of the Police, the Army and the Firefighting brigades. A policy of “evacuate all” was adopted by the Civil Protection. The buildings were re-opened after thorough inspections performed by qualified engineers of the Civil Protection 29.

## Emergency preparedness

According to the statute and regulations of the University l'Aquila, the Rector is the employer as defined by the 81/2008 Law Decree 8. The employer in an Italian organization/institution has major responsibilities and obligations to guarantee health, safety and security in the workplace. The University of L'Aquila Emergency and Safety Organization is shown in Figure 1. In such organigram, the Rector nominates a delegate for emergency and safety management who supervises and coordinates prevention and protection activities. This figure presides the Health and Safety Office - HSO (Area sicurezza ed igiene del lavoro), an office that belongs to the central university administration, which staff is under the direct dependency of the Administrative Director. The HSO deals with the regulations regarding the observance of the Italian Law on workers' safety protection (e.g. biological, chemical, physics and carcinogenic agents protection; and equipment use). The HSO also deals with fire prevention, first aid, evacuation, health surveillance, and the disposal of harmful waste (e.g., radioactive, biological, toxic). The HSO also promotes the training and drills of the university personnel 30. At the operational level, in each primary structure, Colleges, Departments and Services, there is an Health and Safety Advisor, who, among other things, advises the heads of these primary structures on safety and security politics and strategies. The Health and Safety Advisor relates with the HSO and coordinates with other prevention and protection personnel (e.g. fire fighting, first aid, waste management).

**Figure:**
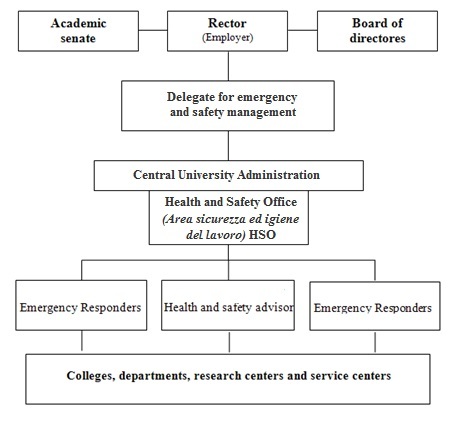
**Fig. 1:** The University of L’Aquila Emergency and Safety Organization

## Recovery policies

After the earthquake, the University gave a rapid response to mitigate the negative effects on teaching and institutional activities. Following is a discussion on goals and measures taken by the University to recover after the earthquake.

**Right to education**: two weeks after the earthquake, the classes restarted in the tensile structures set up by Civil Protection in Coppito. The College of Humanities benefitted of a temporary headquarter located in 3 buildings of the former Juvenile Court in the old town. This solution allowed to maintain the College in the original historical area. Since the academic year 2010/2011 this College was relocated at a former industrial facility in Bazzano, ans it is till waiting for a final destination. The College of Engineering was displaced in temporary campuses located in neighboring municipalities: Avezzano (57 Km from L’Aquila), Lanciano and Pineto, (respectively about 150 and 88 Km far from L’Aquila). During the academic year 2010/2011, Engineering was relocated in a new temporary campus at Campo di Pile (6 Km far from the old headquarter). Few months after the earthquake, the Rector and the professors decided to resume the teaching activities interrupted by the earthquake. E-learning options were activated and the European student exchange Programme Erasmus was encouraged by guaranteeing the extension of their scholarships. Italian colleges, research centers and various private companies offered hospitality in their laboratories to L’Aquila students. The College of Engineering gave its students the opportunity to complete their master thesis in other universities, e.g. Catania, Genova, Parma, Torino, which offered free hospitality in their dormitories to the students of L’Aquila. The damages suffered by the administrative facilities and the houses caused the loss of the academic transcripts, hence the University had to rebuild the students’ careers. The University could recover data only up to March 23, 2009, thus let the students reclaim the exams, they passed between March 24 and April 5, by presenting a written self-declaration, countersigned by the professors who administered the exams.

**Dropouts mitigation**: the University of L’Aquila introduced an incentive mechanism to lower mass migration to other universities and encouraged the recruitment by exempting the following categories of students to pay university fees for the academic year 2009/2010:

- Those enrolling in bachelor or master degree;

- Those renewing the enrollment in bachelor or master degree;

- Those moved from other universities to enroll in bachelor or master degree;

- Those enrolling in bachelor or master to obtain a second degree.

The agreement among the University of L’Aquila, the Regional Directorate of Transport of Abruzzi and the Civil Protection, allowed students enrolled at the University of L’Aquila to apply for free public transport. By mid November 2009, the University of L'Aquila received almost 4000 applications.

**Psychological aid**: in the days after the earthquake, the University opened a new section of the website dedicated to discussions about the disaster. Moreover, a section of the University website commemorates all the students who died in the earthquake (online at: http://www.univaq.it/ricordiamoli/index.html), while the College of Medicine and Surgery with the Rotary Club dedicated a memorial stone to dead students. Furthermore, in the months after the earthquake, each Faculty commemorated the dead students conferring them an honorary degree. On October 27^th^ 2009, the University held a training course on earthquake safety in collaboration with the National Civil Protection, the Italian National Geophysical Institute - INGV and the National Firefighting brigades.

**University facilities management: **after April 6^th^ 2009, the University modified the administrative organization better to cope with the seismic emergency and foster a rapid recovery. New administrative branches/offices were developed: i) Post-Earthquake Rebuilding Planning Office, ii) Earthquake Monitoring Centre, and iii) Aid Management Office. The Post-earthquake Rebuilding Planning Office was responsible to supervise the planning and reconstruction of old and new structures. In detail, this office verified the availability and provenance of external funding (dealing with sponsors), managed the bureaucracy related to construction and reconstruction, planned the final use of structures, and periodically reported to the central university administration the reconstruction progress. The Earthquake Monitoring Centre, is an observatory and research center that studies the earthquake phenomenon from different point of views, collecting data and evidences. This center aims at sensitizing the public on earthquake phenomena. Finally, the Aid Management Office to manage and coordinate donations that came from all over the world.

## Enrollment trends

The office of statistics of the Ministry of Education (MIUR) publishes the annual enrollment data for Italian universities. Trends for first year enrollment and renewed enrollment at the University of L'Aquila were analyzed over the academic years 2004/2005 - 2011/2012. Data were aggregated to analyze trends for the whole university, and split to show differences among the three campuses. Before the earthquake, the Colleges Humanities, Sport Science, Education, and Psychology were located downtown/Centro, the Colleges of Medicine and Surgery, Biotechnology and Mathematics Physics and Natural Science, were located at the Coppito Campus, whereas the College of Engineering and Economics were located at the Roio Campus. Students were distributed as follows: Centro (36.8%), Coppito (33.3%), Roio (29.9%). Over the five years term before the earthquake, the University of L’Aquila had a steady year-by-year increase (Fig. 2). The earthquake of April 2009 produced a relatively small decline (-4.7%) for the first year enrollment, but left unchanged the renewed enrollment for academic year 2009/2010. The lost for the first year enrollment was distributed evenly among the three campuses. The Colleges that reported the most significant decline were Biotechnologies (-46.7%) and Engineering (-25.7%). On the contrary, the Colleges that recorded important boost for the first year enrollment were Economics (46.9%) and Education (26%). As said above, the renewed enrollment for academic year 2009/2010 at the University of L’Aquila was substantially unchanged (Fig. 2). The College that recorded the worst decline in renewed enrollment was Biotechnologies (-15.3%). See Table 1.

**Figure:**
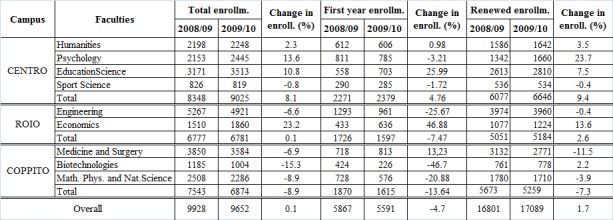
**Table 1:** Change in enrollment (%) between 2008/’09 A.Y. and 2009/’10 A.Y.

**Figure:**
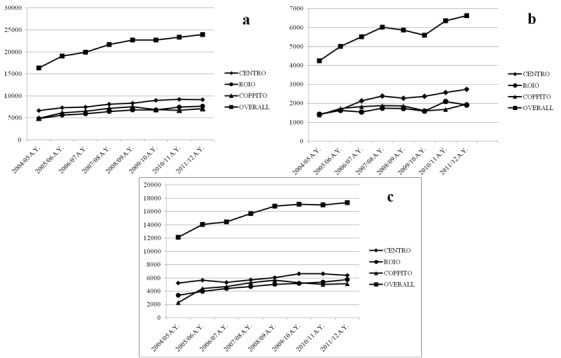
**Fig. 2:** a) Total enrollment ; b) First year enrollment; c) Renewed enrollment

## Questionnaire

**Demographic profile of the respondents. **A total of 110 students answered the questionnaire, 49% of which were females. Their age ranged between 19 and 41 (24.1±3.35). The distribution among the various colleges of the respondents were as follow: College of Mathematics, Physics and Natural Science (28%), Humanities (27%), Medicine and Surgery (22%), Engineering (9%), Psychology (5%), Sport Science (4%), Biotechnologies (3%), Economics and (1%) Education Science (1%). In other words, 53% of respondents were from the Coppito Campus, 37% from Centro, and 10% from the Roio Campus. The percentage from Centro is in line with enrollment data, whilst Coppito and Roio campuses are slightly overestimated and underestimated respectively.

**The role of the Student Associations in pre and post-disaster phases.**

Considering the long lasting seismic swarm that affected the area before the main-shock, interviewed students were asked whether they agreed with the following statement: “Student Associations asked the university administration to verify the structural integrity of the university facilities (classrooms, dorms, and recreational areas).” About 45% of respondents disagreed or totally disagreed, 34% stated otherwise, and about 21% was uncertain. Students were asked whether they thought that Student Associations tried to raise awareness on seismic risk before the earthquake occurred. About 74% of the respondents totally disagreed and 7% disagreed, while 14% agreed. When asked what type of activities were put in place, the following were detailed: leafleting (43%), social networks campaigns (36%), meetings (7%), demonstrations (7%) and drills (7%). The majority of respondents (58%) considered Student Associations a suitable and effective channel to discuss and raise awareness about seismic risk. A similar percentage (59%) affirmed that Student Associations played a pivotal role in reigniting campus life. The totality of the interviewed students claimed that Student Associations did not have a plan to deal with a possible seismic emergency, yet 95% of respondents ignored the emergency plan prepared by the University. Moreover, 75% of the students did not know the existence of external Student Associations (e.g. LARES from Perugia) that came in support to students during the post-emergency phases, and 61% of them ignored the services provided to the University by local Citizen Groups purposely created to deal with the seismic emergency. Among those who knew of the external associations, a small percentage (32%) could specify the role played by these associations during the recovery. Only 2% of the respondents took part to the activities performed by the Student Associations. Results of the psychometric tests are summarized in table 2. The inferential analysis highlighted some patterns: Mann Whitney U test revealed a significant difference in the perception of respondents who knew about Citizen Groups’ activities (Mdn=4) and who did not (Mdn=3) to the statement number 1 (Tab.2), U=537, p<0.05, r=-.23. Similarly, respondents who knew about the external associations (Mdn=4) were more likely to agree to statement number 1 (Tab.2), when compared to those who did not know (Mdn=4), U=498, p<0.05, r=-.19. Regarding the knowledge of the university emergency plan, respondents who knew the plan were more likely to agree to statement number 1 (Mdn=4) - (U=141.5, p<0.05, r=-.22), and statement number 2 (Mdn=4) - U=22, p<0.001, r=-.48, when compared to those who did not know (Mdn=4 and Mdn=4 respectively). Finally, people who knew about Citizen Groups’ activities were almost three times more likely to know about external Student Associations operating during the emergency, χ2(1)=4.06, p<0.05, Φ=.23.

**Table 2: Students' associations:** Results of the psychometric tests (five-items Likert scale statements)

Statements	1. Strongly disagree	2. Disagree	3. Uncertain	4. Agree	5. Strongly agree
1. Student Associations asked the administration to verify the structural integrity of university's facilities	41%	4%	21%	14%	20%
2. Student Associations got active to raise awareness of seismic risk before the earthquake occurred	74%	7%	5%	8%	6%
3. Student Associations are a suitable and effective channel to discuss and create care about seismic risk	22%	6%	14%	20%	38%
4. Student Associations played a pivotal role in the recovery of the campus life	16%	6%	19%	45%	14%

## Discussion

The earthquake of April 6th 2009 hit the facilities of the University of L’Aquila causing the death of 55 students and massive infrastructural damages. Despite the high seismicity of the territory and a long seismic swarm (which begun in October 2008), the University was caught unprepared to cope with the disaster, and the offices accountable for prevention and protection did not disseminate or made known any emergency plan. Nonetheless, because the disastrous earthquake happened at night, the Health and Safety Office, responsible to manage the first response in emergency was not operational (such office had not recovery and business continuity functions). After the disaster, the Rector and the administrative staff worked very hard to mitigate the medium and long-term impact on the University of L’Aquila. Teaching activities were resumed 15 days after the earthquake, thanks to the support of the National Civil Protection, which provided the tensile structures used as classrooms. Moreover, the Declaration of the State of Emergency streamlined the bureaucracy, enabling a fast reorganization of temporary campuses. The Roio campus was relocated in the coastal cities of the “Regione Abruzzo.” The university had to rebuild the students’ careers due to the loss of the academic transcripts (the lack of a data protection and redundancy systems was fairly surprising). Even so, the policies adopted by the University curbed students’ migration; some major tax waivers, and other economic benefits, convinced students not to leave and continue their studies at the University of L’Aquila. The students’ dropout ratio for the academic year following the earthquake was correlated to the dropout of first year enrollment; the renewed enrollment was pretty much unchanged. All that said, it is remarkable to notice that the measures taken by the university administrators were based on an “ad hoc” strategies as no emergency and continuity plans were prepared in advance. As Alexander 31 pointed out, in Italy efforts to avoid the catastrophic consequences of extreme events have almost always been limited to therapeutic actions, rather than on pre-disaster preparedness planning. Indeed, this case study of the University of L’Aquila confirmed such cultural attitude. In regard to the role of Student Associations, perhaps the best proxy to assess their accomplishment, was the 2012 elections to renew the boards of representatives of such associations at the University of L’Aquila. Circa 22% of the students expressed their vote in such election; a good result considering that the average turnout at national level is way less than 20%. This good participation could reflect the trust gained by Student Associations during the emergency. Although, truth to be said, neither these associations planned appropriate disaster risk reduction activities. As with the University administration, the Student Associations at L’Aquila became active during the recovery and restoration activities, but did very little with emergency preparedness and risk awareness programs before the earthquake.

## Conclusions

Italy lacks a culture of disaster prevention and preparedness and relays mostly on adaptive tactics rather than on strategic planning to face emergencies. The case of the 2009 L’Aquila earthquake confirm this state of affairs. The university of L'Aquila lacked a pre-disaster mitigation and recovery plan and only a small portion of students knew some emergency procedures. The information, training and drill activities were not enough to produce an acceptable level of emergency preparedness among students. Indeed, Universities may potentially represent a fertile ground to promote a culture of safety and stop this trend. Greater awareness and involvement should be pushed forward at each level (administrators, faculties, students) to plan in advance for adverse scenarios, and to make steps toward the development of a culture of safety in Italy. Further discussion and research on Universities's emergency preparedness is necessary, also better to clarify the role that Student Associations may play to support and spread a culture of prevention and safety.

## Corresponding Author

Michele Magni: magni.michele@gmail.com

## Data Availability

Data can be accessed at: https://figshare.com/projects/Emergency_preparedness_and_management_at_the_University_of_L_Aquila/17759

## Competing Interest

The authors have declared that no competing interests exist.
